# Enzyme Immunoassay for Measuring Aflatoxin B1 in Legal Cannabis

**DOI:** 10.3390/toxins12040265

**Published:** 2020-04-20

**Authors:** Fabio Di Nardo, Simone Cavalera, Claudio Baggiani, Matteo Chiarello, Marco Pazzi, Laura Anfossi

**Affiliations:** Department of Chemistry, University of Turin, 10125 Turin, Italy; fabio.dinardo@unito.it (F.D.N.); simone.cavalera@unito.it (S.C.); claudio.baggiani@unito.it (C.B.); matteo.chiarello@unito.it (M.C.); marco.pazzi@unito.it (M.P.)

**Keywords:** mycotoxins, food safety, medicinal herbs, competitive immunoassay

## Abstract

The diffusion of the legalization of cannabis for recreational, medicinal and nutraceutical uses requires the development of adequate analytical methods to assure the safety and security of such products. In particular, aflatoxins are considered to pose a major risk for the health of cannabis consumers. Among analytical methods that allows for adequate monitoring of food safety, immunoassays play a major role thanks to their cost-effectiveness, high-throughput capacity, simplicity and limited requirement for equipment and skilled operators. Therefore, a rapid and sensitive enzyme immunoassay has been adapted to measure the most hazardous aflatoxin B1 in cannabis products. The assay was acceptably accurate (recovery rate: 78–136%), reproducible (intra- and inter-assay means coefficients of variation 11.8% and 13.8%, respectively), and sensitive (limit of detection and range of quantification: 0.35 ng mL^−1^ and 0.4–2 ng mL^−1^, respectively corresponding to 7 ng g^−1^ and 8–40 ng g^−1^ ng g^−1^ in the plant) and provided results which agreed with a HPLC-MS/MS method for the direct analysis of aflatoxin B1 in cannabis inflorescence and leaves. In addition, the carcinogenic aflatoxin B1 was detected in 50% of the cannabis products analyzed (14 samples collected from small retails) at levels exceeding those admitted by the European Union in commodities intended for direct human consumption, thus envisaging the need for effective surveillance of aflatoxin contamination in legal cannabis.

## 1. Introduction

*Cannabis sativa* is a plant of the *Cannabaceae* family and is well-known for its content of biologically active chemical compounds, among which the major compounds are delta-9-tetrahydrocannabinol (THC) and cannabidiol (CBD). The flowering or fruiting tops of the Cannabis plant have been controlled under the Controlled Substances Act since 1970 under the drug class “Marihuana” [1].

Cannabis products can be used for medicinal purposes (whether the psychoactive THC or the non-psychoactive CBD, generally referred to as ‘medical cannabis’), in manufacturing (‘industrial hemp’) and for non-medical intoxication (‘recreational or psychoactive cannabis’) [2]. The number of active components found in cannabis and the variety of their effects have also suggested its potential use as a dietary supplement and nutraceutical [3,4]. According to the World Health Organization (WHO), recreational cannabis is the most widely used illicit drug and the most largely cultivated and trafficked worldwide [5].

The therapeutic application of cannabis is increasing across the world [6]. A medicine based on cannabis extract has been approved by the European Medicines Agency [7]. THC for medical application can be administered as capsules, mouth spray or as flowers for making tea. The US Federal Drug Administration (FDA) has approved one cannabis-derived and three cannabis-related drug products [8].

The cultivation and supply of cannabis for industrial use is legal in the European Union since 2013, provided there is a THC content not exceeding 0.2% [9]. In 2018, USA liberalized the production and marketing of hemp, provided that THC content is below 0.3% on a dry weight basis [1].

Advances in liberalization of the use of cannabis for recreational purposes and as dietary supplement and the increase in medical applications are expected to favor the growth of the global legal market of such product in the next years. However, the toxicity to humans of common cannabis contaminants is largely unknown. Due to the ambiguity between legal and illicit production and supply of cannabis products, there is significant lack in the literature regarding the prevalence of cannabis contaminants and of their harmfulness to humans. Contemporarily, progress in the diffusion of cannabis products demands further research in this area, especially because they are used for therapy. [10]

Several classes of contaminants can be present in cannabis including: heavy metals, which are able to bio-accumulate in the cannabis plants [11]; pesticides, (which may also include illegal pesticides because cannabis has been considered illegal for a long time and, therefore, pesticide guidelines or maximal limits for pesticide residues have not been set for this substrate); microbiological contaminants and toxins from microbial overloads, such as ochratoxins and aflatoxins [12,13].

McKernan et al. showed that toxigenic fungi grow on cannabis (especially those producing aflatoxin and ochratoxin) and highlighted the need to investigate the presence of the corresponding mycotoxins in these kinds of sample [14]. Among mycotoxins that can affect cannabis, aflatoxins (AFs) are of utmost concern because of their toxicity and their widespread distribution. AFs are carcinogens, genotoxic and immunosuppressive agents [15]. In particular, aflatoxin B1 (AFB1) is the most recurrent and carcinogenic of the aflatoxins, and it is well documented to be a causative agent of hepatocellular carcinoma as well as growth suppression, immune system modulation, and malnutrition [16,17]. AFB1 is produced by fungi of the *Aspergillus* genus, namely *Aspergillus flavus* and *Aspergillus parasiticus*.

A. flavus is ubiquitously found in soil and contaminates a wide range of the world’s crops. After establishing the plant as a host, the fungus produces aflatoxins, including AFB1. Fungal growth can occur on crops at any point in the pre- or post-harvest stage. Additionally, high temperatures and humidity favor fungal growth, so carelessness of storage conditions favors a large amount of AFB1 contamination occurring during storage [18].

The lack of regulations and the prevailing illegal production, storage and consumption of cannabis have meant a general unavailability of controls on its safety, including the absence of methods to monitor contamination. In this work, a rapid and sensitive enzyme immunoassay for measuring AFB1, primarily developed to monitor the presence of the toxin in eggs [19], was adapted for detecting AFB1 in cannabis products. Although several accurate and sensitive immunoenzymatic kits for AFB1 detection are available on the market, the indiscriminate use of immunoassay kits originally developed and validated for application in specific matrices to monitor AFB1 in very different materials should be carefully evaluated. Therefore, samples of cannabis derivatives (inflorescence and leaves) legally sold under the requirement of THC content lower than 0.2% were collected in small retail outlets in Torino (Italy). The enzyme immunoassay was modified in order to comply with the effect of the herbaceous matrix and the modified assay was in-house validated. A chromatographic-tandem mass spectrometry method was also developed to confirm accuracy of the enzyme immunoassay. Finally, the sensitive enzyme immunoassay was used to measure AFB1 contamination in 14 samples of cannabis products.

## 2. Results

### 2.1. Enzyme Immunoassay Adaptation to AFB1 Detection in Cannabis Products

Extraction of the aflatoxin B1 from cannabis leaves and seeds was carried out by partitioning in 80% methanol, as previously reported for AFB1 extraction from several kinds of medical plants [20,21].

The enzyme immunoassay used in this work was initially developed for measuring aflatoxins in eggs [19] and consisted of a direct competitive immunoassay, in which a polyclonal antibody raised against aflatoxin M1 linked to bovine serum albumin (BSA) (antiM1-pAb) was adsorbed onto the polystyrene of microplate wells. The target compound (AFB1) and the enzyme probe (AFB1 linked to horse radish peroxidase, AFB1-HRP) competed for binding to the anchored antibody. After removing unbound fractions by washing the plate, the signal generated by the enzyme was developed and measured. The time required to complete the analysis was 30 min. In previous work, we also produced a second polyclonal antibody using AFB1 linked to BSA as the immunogen (antiB1-pAb). The antiB1-pAb showed higher selectivity towards AFB1 compared to the antiM1-pAb and was used in this work. Therefore, optimal AFB1-HRP and antiB1-pAb concentrations were defined ex-novo through the checkerboard titration approach. Other assay parameters were also re-evaluated. In particular, AFB1-HRP and antiB1-pAb concentrations and time of reactions were decided upon providing a signal of the blank of approximately 1.5 UA and an IC_50_ of the calibration curve below 1 ng mL^−1^. Other parameters were defined based on minimizing matrix effect. Hence, extracts were fortified with known concentrations of AFB1 and the relative matrix effect (ME%) was calculated as follows:

ME% = (AFB1 measured in the fortified extract − AFB1 measured in the non-fortified extract) / AFB1 added × 100 [22].

As the scope of the re-optimization of the enzyme immunoassay was intended for coping with new interference in AFB1 quantification due to the specific composition of the cannabis matrix, recovery was measured by fortifying the extract, which included potential interfering substances deriving from the sample.

A modification of the pristine protocol was considered for statistically significant improvement of the obtained ME% rate.

Two samples (representative of leaves and inflorescence) collected in a small local retail outlets were extracted and, using the methanolic extracts fortified with AFB1, the following parameters were studied: (1) dilution of the methanolic extract with water; (2) volume of the diluted extract to be added to the reaction well; (3) time for the immuno- and enzymatic reactions; (4) nature of the buffer for AFB1-HRP dilution; and (5) composition of the washing solution.

In particular, we observed that a precipitate formed when the methanolic extracts were diluted with water; however, filtration and centrifugation to remove the particulate matter caused a dramatic loss of the toxin, measured by recovery values below 50%. Then, the raw suspension was diluted 1 + 1 and added directly to the wells. Higher dilution rate (1 + 3) decreased the sensitivity of the assay (because of sample dilution) without increasing recovery rates, while using the undiluted extract produced a strong matrix effect evidenced by a large overestimation of the AFB1. For avoiding excessive matrix interference, the sample volume was reduced to one half (further reducing sample volume was ineffective for increasing recovery and halved the sensitivity). The pH of the buffer used for the immunoreaction and of the washing solutions was also modified in order to obtain satisfying recovery rates. Specifically, lowering the pH of both solutions to 5.0 allowed us to suppress most of the matrix interference. On the contrary, modification of composition (salts and additives) of buffers did not allow us to significantly improve recovery rates (Figure S1). Finally, the time of reactions was defined to limit the overall time required for completing the analysis while providing a signal of the blank that was measured with acceptable precision (>1 UA). The total time for the analysis was 40 min, which is quite low for microplate-based immunoassays and acceptable for the intended use as a first-level screening analysis. The experimental conditions considered in the study and the protocol optimized for AFB1 detection in cannabis are shown in Table 1. Several parameters of the pristine protocol needed to be modified to achieve acceptable recovery rates in the detection of AFB1 in cannabis products instead of in egg yolk. This finding pointed out that the use of commercial kits originally intended for specific applications to different commodities without modifications can lead to inaccuracy and should be discouraged.

**Figure 1:**
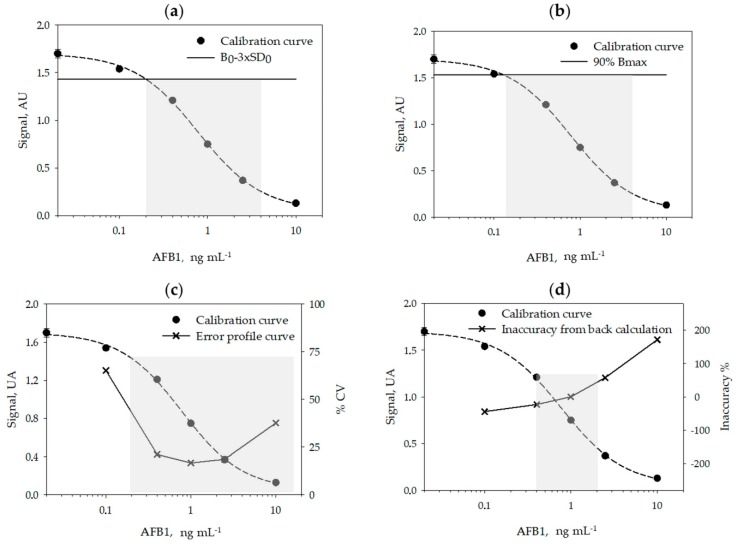
The mean calibration curve obtained by averaging results from 6 individual curves carried out on six days. The limit of detection and the quantification interval calculated according to different methods are shown by grey areas: (**a**) signal-to-noise method [22,23], (**b**) IC_10/20-80_ method [24,25,26,27], (**c**) error profile method [28], and (**d**) back calculation method [29].

A typical calibration curve for measuring AFB1 obtained in the optimized conditions is shown in Figure 1.

### 2.2. Analytical Figures of Merits of the Enzyme Immunoassay

Using 6 calibration curves, generated on different days and by using 6 calibrators measured in duplicate on each day, we studied the reproducibility of the calibration (Table 2) and calculated the limit of detection (LOD) and the range of quantification (ROQ) of the assay (Table 3 and Figure 1). Signals recorded on each day were normalized by the signal of the calibrator containing no AFB1 (B_0_). The LOQ and ROQ were estimated according to four methods, variously applied to competitive immunoassays: the signal-to-noise method [22,23], the IC_10/20–80_ method [24,25,26,27], the error profile method [28], and the back-calculation method [29].

The calibration parameters were acceptably repeatable within different analytical sessions and days. The limit of detection varied depending on the method used for its estimation between 0.12 ng mL^−1^ (B_max_ inhibition) and 0.35 ng mL^−1^ (back-calculation method). The quantification range also varied depending upon the method used to calculate it. Especially, the back-calculation method gave the narrower interval (0.4–2 ng mL^−1^) while according to the error profile method, the quantification range spanned from 0.2 to 14 ng mL^−1^ (Figure 1). The LOD varied among methods by a factor of 3 and the ROQ approximately by one order of magnitude. Whatever the method, the enzyme immunoassay showed high sensitivity.

Selectivity towards other mycotoxins was measured by calculating the cross-reactivity (CR), defined as follows: CR% = IC_50_ AFB1/IC_50_ mycotoxin × 100 (Table S1). The selectivity trend was similar to the one observed previously for the same antibody [16]. In details, other compounds in the class of aflatoxins showed a certain degree of cross-reactivity, which ranged from 2.0% (AFM1) to 25.3% (AFG1). Other mycotoxins with unrelated structures (i.e., ochratoxin A, zearalenone and fumonisins) did not interfere at all.

### 2.3. Measuring AFB1 in Cannabis Products by the Enzyme Immunoassay

The trueness of the assay was studied by recovery experiments. Two cannabis samples (#JA, made of leaves, and #DI comprising inflorescence) were analyzed directly and after fortification of the raw sample (10 and 20 ng/g of AFB1). Apparently, sample #DI contained AFB1 (9.6 ng g^−1^), while sample #JA showed an apparent AFB1 content of 2.8 ng g^−1^ (corresponding to 0.28 ng mL^−1^ in the extract). This value was below the LOD estimated by the back-calculation method, while exceeding those calculated by the other methods. Sample #JA was then diluted 1 + 1, 1 + 3, and 1 + 7 with the extraction solvent and analyzed again. We expected that sample dilution would produce a proportional signal increase. On the contrary, signals were randomly scattered. We conclude that AFB1 content of sample #JA was below the detection limit of the assay; therefore, we assumed the LOD calculated by the back-calculation method as the most reliable for determining AFB1 in cannabis samples. According to the assignment of sample #JA as containing undetectable amounts of AFB1, satisfactory recovery rates (83–113 %, Table S2) were obtained for both samples.

The reproducibility of the enzyme assay was evaluated by measuring one sample in six replicates within the same day (intra-assay repeatability) and five samples in duplicates on two different days (inter-assay variability). The intra- assay relative standard deviation (RSD %) and the mean of inter-assay RSD% were 11.8% and 13.8%, respectively.

### 2.4. Liquid Chromatography Tandem Mass Spectrometry Determination of AFB1 in Cannabis Products

To validate the enzyme immunoassay, a HPLC-MS/MS method for measuring AFB1 in cannabis products was developed in-house by adapting the method of Zheng et al., previously reported for the detection of major aflatoxins in medicinal herbs [30]. The method of Zheng et al. involved the analysis of the crude herbal extract without purification or pre-concentration and allowed the differentiation of various aflatoxins. The separation was obtained by a gradient elution in reverse phase liquid chromatography and the detection was in the single reaction monitoring (SRM) mode. To comply with matrix interference, AFM1 was used as the internal standard, provided that AFM1 forms from the animal metabolism and then its presence in herbal extract could be excluded. The linearity of the calibration was confirmed between 5–40 ng mL^−1^ (y = 0.56x − 0.67, r2 = 0.992, Figure S2) and the LOD and LOQ were calculated as 1.8 and 5.8 ng mL^−1^ (corresponding to 18 and 58 ng g^−1^ in the sample), respectively. The limit of detection of the HPLC-MS/MS method was five to ten times higher than the one calculated for the enzyme immunoassay (depending on the method used to calculate this last). The poor sensitivity compared to chromatography coupled to mass spectrometry [31] was due to the fact that we analyzed the crude extracts without applying any clean-up or pre-concentration and that we did not optimize the method. To evaluate matrix interference four samples were analyzed by the HPLC-MS/MS method. All samples contained AFB1 below the limit of detection of the method. The extracts from four samples (two extracts for each sample,) were then fortified with 10 ng mL^−1^ of AFB1. Relative matrix effect values for fortified samples ranged from 81 to 123% with a certain variability also among duplicate samples (Table 4).

### 2.5. Method Comparison: Enzyme Immunoassay and HPLC-MS/MS

To further confirm that the enzyme immunoassay was not affected by the interference of the matrix and by its intrinsic variability (leaf, flowers, seeds and other parts of the cannabis plant were occasionally present in the samples collected in small retail outlets), four samples were divided into sub-samples (two sub-samples were generated for each sample) and extracted and analyzed on different days. As observed for the HPLC-MS/MS validation, again certain variability between sub-samples was observed (Table 4).

In parallel, a further total of 10 samples was extracted and analyzed directly after fortifying the extracts with 10 ng mL^−1^ of AFB1 by the in-house-developed HPLC-MS/MS method and by the enzyme immunoassay (fortified extracts were analyzed by the enzyme immunoassay after a 1:10 dilution in the extraction solvent to comply with the ROQ). All samples resulted containing AFB1 below the limit of detection of the HPLC-MS/MS method, while according to the enzyme immunoassay 50% of samples were contaminated above the LOD. The mean AFB1 content was measured to be 12.3 ng g^−1^ and the contamination level varied between 8.6 and 17.7 ng g^−1^ (Table 4).

**Figure 2:**
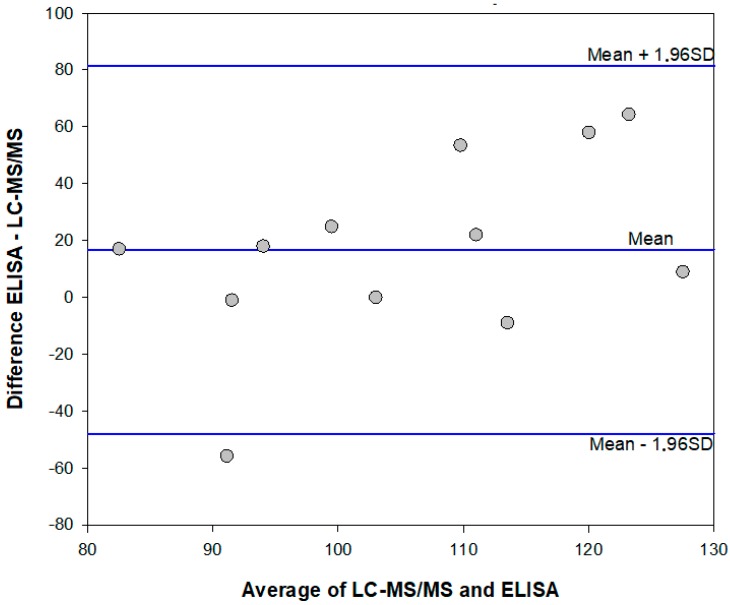
Bland-Altman plot for comparing the enzyme immunoassay and HPLC-MS/MS methods to measure AFB1 in cannabis products. Data are randomly scattered, with a positive bias of +16, representing the tendency of the enzyme immunoassay to overestimate AFB1 compared to the HPLC-MS/MS method.

The mean ME% calculated for fortified extracts were 108% (78–136%) and 99% (74–123%) for the enzyme immunoassay and the HPLC-MS/MS method, respectively. Results which agreed were obtained in the two analytical methods, although the enzyme immunoassay showed a tendency to overestimate AFB1 contamination in comparison to the HPLC-MS/MS method. (Figure 2 and Table 4).

## 3. Discussion

A rapid, accurate and sensitive enzyme immunoassay was established for the measurements of AFB1 in cannabis products, based on previously developed bio reagents. The re-evaluation of assay parameters and particularly of the pH of the buffers and the washing solution allowed us to adapt the assay to the novel matrix and to mitigate the influence of the large variability in the composition of extracts from different part of the cannabis plant. To comply with possible variability of the matrix, a prudential limit of detection was decided, which was calculated from the inaccuracy of repeated calibration curves [29] and validated by dilution and recovery experiments on two cannabis samples. Actually, the limit of detection (LOD) and the range of quantification (ROQ) are variously defined for immunological-based assays, in particular for competitive immunoassays, where the signal is inversely (and not linearly) correlated to the concentration of the target. Sometimes, the signal-to-noise ratio method [22,23] is used to calculate the LOD, which is then assumed as the concentration of the analyte that corresponds to the signal of the standard 0 (B_0_) minus two or three standard deviation of the standard 0. However, this method has some limitations when applied to non-linear curve fitting. As an alternative, especially suitable for competitive immunoassays in which data are fitted by the four parameter logistic model (4-PL), a certain level of inhibition of the maximum binding (B_max_) is considered to estimate the LOD and ROQ [24,25,26,27,32,33]. The inhibition levels most frequently considered for the purpose are 90% for estimating the LOD, and 85%–15% [32,33] or 80–20% [24,25,26,27] for the ROQ, respectively. The rationale beyond this approach is represented by the fact that the typical standard curve of competitive immunoassay*s* has a sigmoidal shape and the upper and lower parts of the curve are strongly imprecise. However, the inhibition levels are, in some way, arbitrarily defined. A more robust identification of significant inhibition levels is based on the use of the error profile curve (also called precision profile). In this method, the relative standard deviation (RSD %) of repeated experiments is calculated for various concentrations of the analyte (typically for calibrators) and plotted towards the calibrators’ concentrations. The ROQ and LOD are defined as the interval of concentrations that can be measured with a certain precision [28,34]. However, the level of acceptable imprecision is debated. Some authors have 30% and 10% for estimating the LOD and ROQ, respectively [30], while others considered 50% as the maximum acceptable imprecision [32]. In addition, modelling precision profile is complicated and discourages the application of this criterion. A concept similar to using the precision profile is the back-calculation method, in which the concentration of the calibrators is estimated by the fit of the curve and the interval of quantification is defined as the concentrations estimated with an acceptable accuracy (±20%) [29,34]. The limit of detection is calculated as the lower concentration that provides inaccuracy below 25% [29].

In this work, we used repeated calibration curves to estimate LOD and ROQ according to the four approaches described above. The values obtained for the LOD varied approximately by a factor of three depending on the approach applied; the IC_10/20-80_ method provided the lowest value (0.12 ng mL^−1^) while the highest value (0.35 ng mL^−1^) was calculated according to the back-calculation method. The quantification range varied also upon the method used to calculate it and to a larger extent than the LOD. The back-calculation method provided the narrower interval (0.4–2 ng mL^−1^) and the error profile method the largest interval (0.2 to 14 ng mL^−1^). From a theoretical point of view, the error profile and the back-calculation approaches are the more robust; however, they require several experiments and complicated mathematical modeling. The signal-to-noise ratio allows the obtaining of a reasonable compromise, although it is based on the assumption of the linear dependency of the signal on the analyte concentration, which is not realistic for ligand-binding assays. The simplest method to calculate the LOD and ROQ is that based on defining levels of inhibition of B_max_. In this case, it is sufficient to fit the data and interpolate the values corresponding to IC_10_, IC_20_ and IC_80_. The simplicity of the calculation explains the success of the approach; however, the enzyme immunoassay here reported produced a significantly lower LOD compared to other approaches. Moreover, the IC_10_ limit was not robust when considering the capability of the assay for detecting AFB1 in real samples. The LOD calculated form other methods allowed us to reliably measure AFB1 in the extract from different cannabis products. The comparison suggests some precaution in comparing different competitive immunoassays where the analytical performance was calculated differently.

The estimated LOD for measuring AFB1 in cannabis leaves and flowers (3,5 ng g^−1^) was higher than that recently reported by Narváez et al. [31]. However, the ultra-high sensitivity was reached by using ultra-high performance liquid chromatography coupled to high resolution tandem mass spectrometry and requested a preliminary clean-up of the extracts. The enzyme immunoassay was applied to extracts without additional treatment and required cost-efficient equipment and very limited training of personnel to be operated, thus allowing wide applications in low resource settings and for the affordable monitoring of the safety of the cannabis product, especially considering recreational use and employment as food supplement. The limited number of samples analyzed in this work does not permit us to draw conclusions about the risk of AFB1 contamination in cannabis products legally sold in Italy; however the 50% of samples we analyzed, showing AFB1 contents above the detectable level and above the maximum limit admitted for commodities intended for direct human consumption [35], shed light on the need for increasing controls and, more generally, investigating the level of contamination from mycotoxins of such products. These preliminary results also suggest implementing appropriate surveillance of aflatoxin contamination of cannabis products intended for medical uses. In addition, specific analytical methods to measure other toxic metabolites in cannabis products should be developed in order to effectively protect consumers’ health.

## 4. Materials and Methods

### 4.1. Reagents and Apparatus

Bovine serum albumin (BSA), 3,3′5,5′-tetramethylbenzidine liquid substrate (TMB), and Aflatoxin B1, aflatoxin M1, aflatoxin B2, aflatoxin G1, aflatoxin G2, ochratoxin A (OTA), deoxynivalenol (DON), fumonisin B1 (FB1), and zearalenone (ZEA) standard solutions were purchased from Sigma Aldrich (Merck, Darmstadt, Germany). Methanol (HPLC grade), microplates and all other chemicals were obtained from VWR International (Milan, Italy). Rabbit polyclonal antibodies directed towards aflatoxin B1 (anti-AFB1) and aflatoxin B1 conjugated to horse radish peroxidase (AFB1-HRP) were prepared in the laboratory as described in [16]. Optical density at 450 nm was measured by a Multiskan microplate reader (ThermoScientific, Waltham, MA, USA). Extract were centrifuged in a refrigerated centrifuge (BR, Juan, France).

### 4.2. Competitive Direct ELISA

The assay was carried out as described previously, with minor modifications to assure optimal detectability, as follows.

The immunoreactive wells were prepared by adsorbing overnight anti-AFB1 antibodies diluted in carbonate/bicarbonate buffer (pH 9.6). After washing with 0.05% Tween 20, uncoated well surface was saturated with 0.5% BSA dissolved in phosphate buffer supplied with 0.15M NaCl and 0.05% Tween 20 (PBST_BSA) for 1 h at room temperature, followed by three washings with 0.05% Tween 20.

Calibration curves were generated by mixing 150 µL of AFB1-HRP (0.05 μg mL^−1^) in PBST_BSA and 50 µL of AFB1 standards diluted in aqueous methanol (40%) at concentrations ranging from 0 to 10 ng mL^−1^. After 15 min incubation in immunoreactive wells, unbound reagents were removed by five washings with PBST. Color due to TMB oxidation was stopped after 25 min incubation by adding sulphuric acid (2M) and measured at 450 nm. For cannabis samples, extracts prepared as described below were directly added to wells instead of AFB1 standards. All standards were measured in duplicate.

Unknown sample concentrations were determined by interpolation on the calibration curve, where the signal was plotted against the analyte concentration. For each experiment, a calibration curve was determined by a nonlinear regression analysis of the data using the four-parameter logistic equation.

Reproducibility of the calibration was evaluated by comparing curves obtained on six days. On each day, the normalized signal (S/S_0_, %) was calculated as the signal produced by each calibrator (S) divided by the signal of the standard 0 (S_0_). Inter-day reproducibility was calculated as the coefficient of variation of the mean of normalized signals for each AFB1 level. In addition, percentage error of concentration was defined by back-calculating concentrations at each AFB1 level on each day and then considering the coefficient of variation of back-calculated concentrations.

The limit of detection and working interval of concentrations were estimated from the calibration curve obtained by averaging six individual curves according to different methods from the literature [22,23,24,25,26,27,28,29].

In particular, for the signal-to-noise method, the mean value and the standard deviation of the calibrator “0” (blank) were calculated from 12 replicates (6 days × 2 replicates on each day). The signal-to-noise ratio was set at 3 and the LOD was calculated as the concentration corresponding to the blank minus three standard deviation of the blank. The ROQ was estimated from the curve as the interval that can be considered as approximatively linear, even if a competitive dose-response curve is intrinsically non-linear. The IC_10/20–80_ method estimates the LOD as the 10% inhibition of the maximum binding (B_max_), while the ROQ is represented by values comprises between 20% and 80% of B_max_ inhibition. In this case, B_max_ is obtained from the 4-PL fit of data.

The error profile and the back-calculation methods encompass the measuring of the coefficient of variation and the inaccuracy at different levels of the target and plotting them towards the levels. LOQ and ROQ are defined accordingly with acceptable imprecision and inaccuracy. The error profile curve and the inaccuracy curve were also generated from repeated measurements of the calibrators (*n* = 12). The error profile curve was obtained by plotting the RSD% of calibrators towards their concentration, while the inaccuracy was obtained from the back-calculation of calibrator concentrations obtained by the 4 PL-fit compared to the true value for each calibrator.

### 4.3. Cross-Reactivity Study

Calibration curves as described above were generated for other aflatoxins (AFG1, AFB2, AFG2, and AFM1) and unrelated mycotoxins (ochratoxin A, deoxynivalenol, fumonisins B1, and zearalenone). The same protocol was applied; however, a larger concentration range was investigated for unrelated mycotoxins (0–100 ng mL^−1^).

Relative cross-reactivity was calculated as follows:

CR% = (IC_50_ AFB1/IC_50_ mycotoxin) × 100

where IC_50_ is the mycotoxin concentration which causes 50% inhibition of the maximum observed signal.

### 4.4. Samples and Sample Preparation

Fourteen samples of legal cannabis were purchased in small retail outlets in Torino (Italy) during the period January-March 2019. Samples were roughly minced, accurately weighted (0.2 g) and extracted with 2 mL of aqueous methanol (80%) by vortex mixing for 2 min and centrifuging at 5000× *g* for 15 min (4 °C). Supernatants were diluted 1 + 1 with water and analyzed by the direct competitive ELISA. Depending on sample availability, 1-3 sub-samples were separately weighted and extracted. Extracts were analyzed in quadruplicate.

Two samples that did not show any detectable residues of aflatoxins were taken as the blank for recovery experiments. Fortified samples were prepared by adding 10, and 20 ng g^−1^ of AFB1, to the minced samples, leaving overnight under a hood for drying the solvent and homogenizing.

### 4.5. Liquid Chromatography Coupled to Tandem Mass Spectrometry Detection of AFB1

The chromatographic separation was achieved by an Accela System (ThermoScientific, Waltham, MA, USA) and using a kinetic XB-C18 column (150 mm × 4.6 mm; 5 µm form Phenomenex, Torrance, CA, USA). Ammonium acetate (4 mmol L^−1^) and 0.1% formic acid (A) and methanol (B) were used as the mobile phase. Gradient elution was programmed as follows: 5 min isocratic elution at 40% B, linear increase up to 80% in 15 min, then up to 100% in further 5 min and finally, isocratic elution at 100% for further 5 min. The total run time including re-conditioning was 30 min. Detection was obtained by the SRM method on a LCQ Fleet (ThermoScientific, Waltham, MA, USA), equipped with the electrospray source operating in positive mode. Transitions followed for AFB1 and AFM1 are detailed in Table S3. For quantification, the calibration curve was built by plotting thee area of AFB1 peak divided by the area of AFM1 peak towards AFB1 concentrations (Figure S1). The LOD and LOQ were calculated from equations:

LOD = (3 × SD)/m

LOQ = (10 × SD)/m

## Figures and Tables

**Table 1:** Protocol for the detection of aflatoxin B1 (AFB1) in cannabis leaves and flowers.

Variable	Pristine Protocol Deduced from [19]	Conditions Considered in This Work ^1^
volume of standard/sample	100 µL	25, **50**, 100 µL
Dilution factor of methanol extract	1 + 1	1 + 0, **1 + 1**, 1 + 3
Buffer for diluting AFB1-HRP	PBST pH 7.4	**PBS/T pH 5.0**, 6.0, 7.4 MES/T pH 6.0 phosphate/citrate/T pH 6.0 Tris/T pH 7.4, 8.5
Washing solution composition	0.3 M NaCl + Tween 20	0.05% Tween 20, 0.3 M NaCl/T, PBS/T pH 7.4, **PBS/T pH 5.0**
Time of reaction	15′ + 15′	15′ + 15′, 25′ + 15′, **15′ + 25′**

^1^ The conditions selected for the enzyme immunoassay are highlighted in bold.

**Table 2:** Parameters of the calibration curve fitting. Mean ± SD of the parameter were calculated from 6 curves obtained on different days. The fit was obtained from 6 calibrators, including the blank. Each calibrator was measured in duplicate on each day. The 4-parameter logistic model was used for curve fitting.

Parameter	Mean ± SD
B_max_ (UA)	1.7 ± 0.2
B_min_ (UA)	0.08 ± 0.01
IC_50_ (ng mL^−1^)	0.8 ± 0.1
Slope	−1.26 ± 0.05

**Table 3:** Analytical figures of merits of the enzyme immunoassay for measuring AFB1 estimated according to different definitions of limit of detection (LOD) and range of quantification (ROQ) reported in the literature [22,23,24,25,26,27,28].

Method	Definition of LOD	LOD (ng mL^−1^)	Definition of ROQ	ROQ (ng mL^−1^)	Ref.
Signal-to-noise ratio	B0–3sd0	0.2	linearity (y vs log x)	0.2–2.5	[22,23]
B_max_ inhibition	IC_10_	0.12	IC_20_–IC_80_	0.15–4	[24,25,26,27]
Error profile	RSD% = 50%	0.2	RSD% = 50%	0.2–14	[28]
Back-calculation	Inaccuracy = 25%	0.35	Inaccuracy = 20%	0.4–2	[29]

**Table 4:** AFB1 content in cannabis products from small local retails as measured by the enzyme immunoassay and by the HPLC-MS/MS and matrix effect for sample extracts fortified with 10 ng mL^−1^ of AFB1.

Sample Id #	Enzyme Immunoassay		HPLC-MS/MS	
	AFB1 ± SD (ng g^−1^)	ME ^a^ (%)	AFB1 ± SD (ng g^−1^)	ME ^a^ (%)
NH-1	12.1 ± 0.9	86	<LOD ^c^	74
NH-2	14.8 ± 2.6	111	<LOD ^c^	91
GA-1	<LOD ^b^	118	<LOD ^c^	81
GA-2	8.7 ± 0.1	117	<LOD ^c^	96
WA-1	<LOD ^b^	104	<LOD ^c^	103
WA-2	<LOD ^b^	116	<LOD ^c^	123
EJ-1	<LOD ^b^	102	<LOD ^c^	123
EJ-2	<LOD ^b^	103	<LOD ^c^	118
VW	13.8 ± 0.2	118	<LOD ^c^	99
AF	<LOD	78	<LOD ^c^	119
GS	<LOD	136	<LOD ^c^	91
DP	13.4 ± 1.8	86	<LOD ^c^	92
BE	11.5 ± 1.1	126	<LOD ^c^	83
LE	<LOD	98	<LOD ^c^	85
AH	<LOD	116	<LOD ^c^	100
JA	17.7 ± 0.2	- ^d^	- ^d^	- ^d^
DI	9.7 ± 0.9	- ^d^	- ^d^	- ^d^
SO	8.6 ± 0.4	- ^d^	- ^d^	- ^d^

^a^ ME value was calculated as (AFB1 measured in the fortified sample—AFB1 measured in the raw sample)/AFB1 added*100. For the enzyme immunoassay, fortified extracts were diluted 1:10 before analysis. ^b^ The value obtained from the back calculation method (3.5 ng g^−1^) was considered. ^c^ LOD for the HPLC-MS/MS method was 18 ng g^−1^. ^d^ Not determined.

## Supplementary Materials

The following are available online at https://www.mdpi.com/2072-6651/12/4/265/s1: Figure S1: Optimization of the composition of AFB1-HRP diluent and of the washing solution, Figure S2: Calibration curve for the HPLC-MS/MS method to measure AFB1 in cannabis products, Table S1: Cross-reactivity of the enzyme immunoassay towards mycotoxins, Table S2: Recovery rates for two cannabis samples fortified with AFB1 and analyzed by the enzyme immunoassay, Table S3: SMR transitions for AFB1 quantification in cannabis products.
