# Supplementary material for: Enzyme Immunoassay for Measuring Aflatoxin B1 in Legal Cannabis
Source: Toxins (Basel). 2020 Apr 20;12(4):265. doi: 10.3390/toxins12040265 (PMC7232199; doi:10.3390/toxins12040265)
Supplement: Supplementary file 1 [file toxins-12-00265-s001.pdf]

Supplementary Materials: Enzyme immunoassay for measuring aflatoxin B1 in legal cannabis

Fabio Di Nardo, Simone Cavalera, Claudio Baggiani, Matteo Chiarello, Marco Pazzi and Laura Anfossi

Table S1. Cross-reactivity of the enzyme immunoassay towards mycotoxins.

Mycotoxin	CR%
AFB1	100
AFB2	15.8
AFG1	25.3
AFG2	4.3
AFM1	2,0
ochratoxin A	<0,1
deoxynivalenol	<0,1
zeralenone	<0,1
fumonisins B1	<0,1

Table S2. Recovery rates for two cannabis samples fortified with AFB1 and analyzed by the enzyme immunoassay.

Sample id #	Fortification Level (ng g^{-1})	AFB1 ± SD (ng g^{-1})	Recovery (%)
JA	0	<LOD [a]	-
	10	8.8 ± 0.2	88
	20	20.6 ± 0.4	103
DI	0	9.7 ± 0.6	-
	10	16.6 ± 0.4	83
	20	33.6 ± 1.3	113

[a] The value obtained from the back calculation method (0.35 ng ml^{-1}) was considered.

Table S3. SMR transitions for AFB1 quantification in cannabis products.

Analyte	Retention Time	Precursor ion	Product Ion
AFB1	16.1 ± 0.1	313 $[M+H]^+$	285 $[M+H – CO]^+$
AFM1	12.2 ± 0.1	329 $[M+H]^+$	301 $[M+H-H_2O]^+$

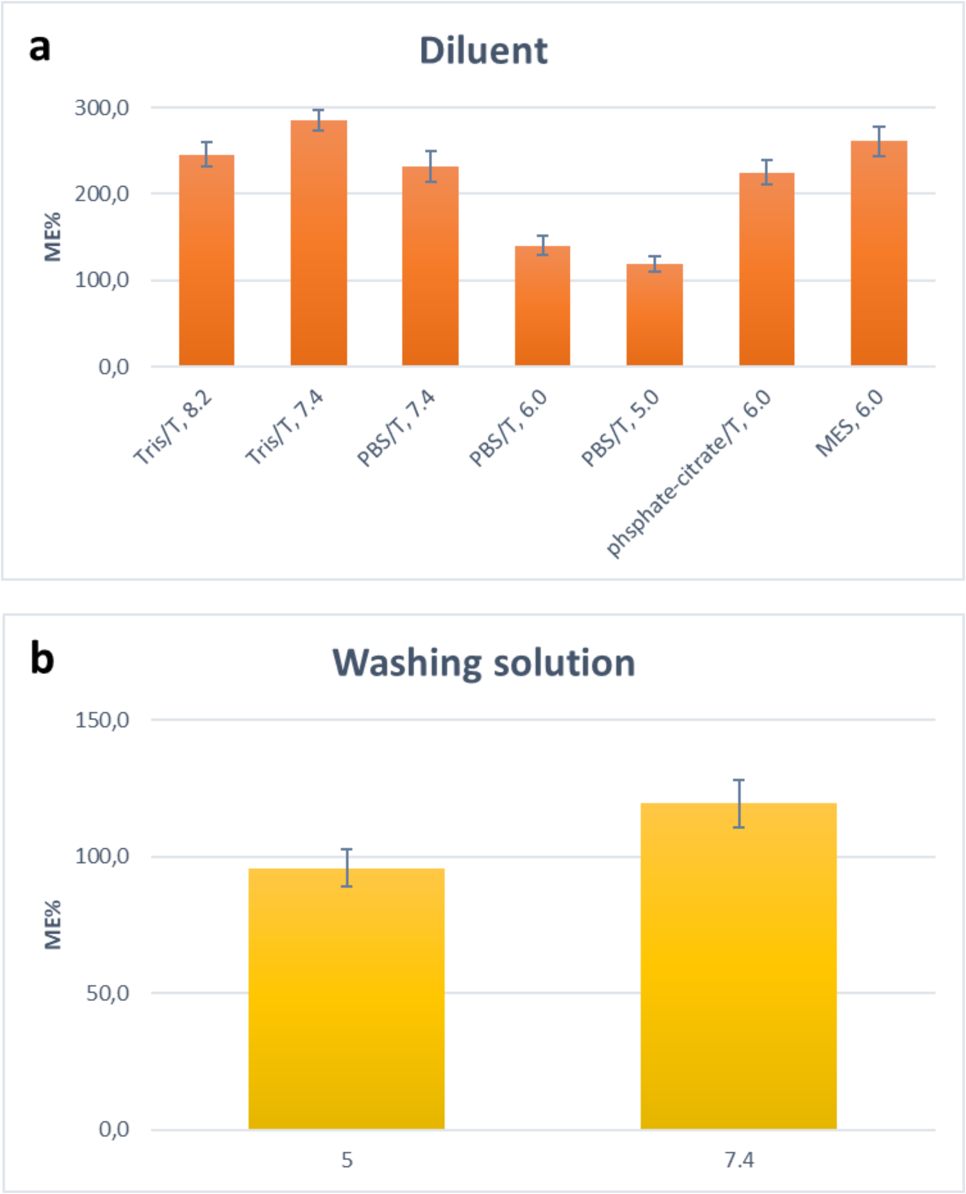

Figure S1. Matrix effect calculated from a fortified extract of cannabis flower when AFB1 was estimated by using different buffers as the AFB1-HRP diluent (**a**) and by removing unbound fractions by washing solutions with different pH (**b**).

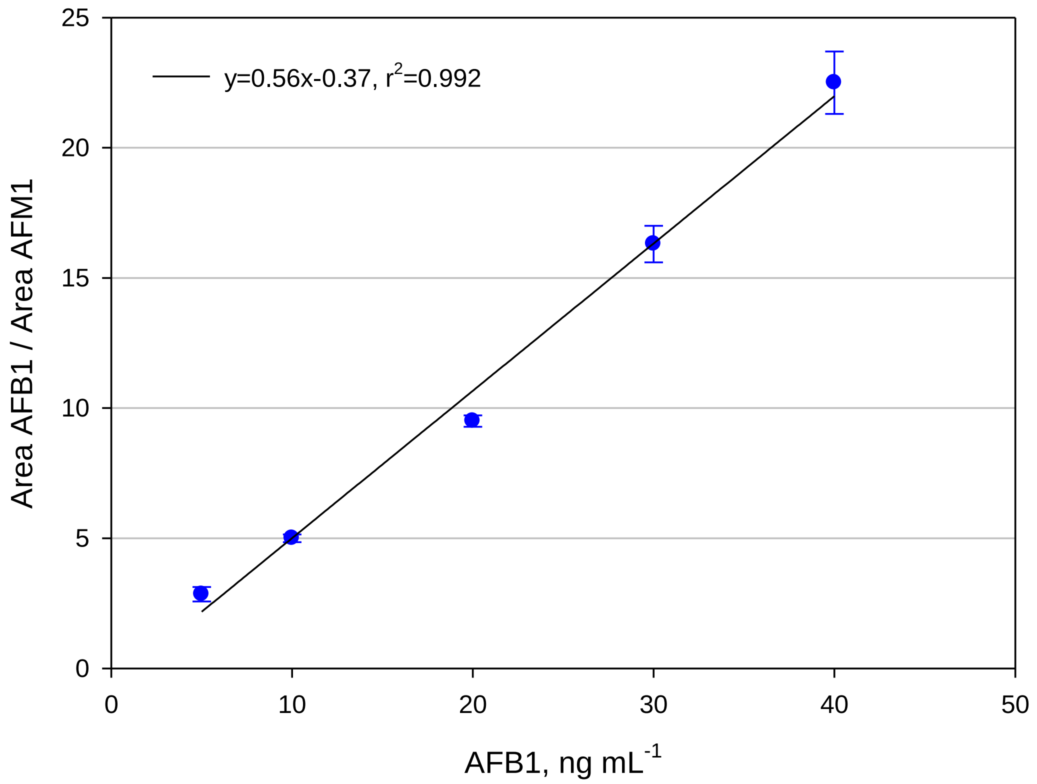

Figure S2. Calibration curve for the LC-MS/MS method to measure AFB1 in cannabis products. Bars represent standard deviations of three replicate measurements.
